# Synthesising 2D Video from 3D Motion Data for Machine Learning Applications

**DOI:** 10.3390/s22176522

**Published:** 2022-08-29

**Authors:** Marion Mundt, Henrike Oberlack, Molly Goldacre, Julia Powles, Johannes Funken, Corey Morris, Wolfgang Potthast, Jacqueline Alderson

**Affiliations:** 1 UWA Minderoo Tech & Policy Lab, Law School, The University of Western Australia, Crawley, WA 6009, Australia; 2 Institute of General Mechanics, RWTH Aachen University, 52062 Aachen, Germany; 3 Institute of Biomechanics and Orthopaedics, German Sport University Cologne, 50933 Cologne, Germany; 4 School of Human Sciences, The University of Western Australia, Crawley, WA 6009, Australia; 5 Sports Performance Research Institute New Zealand (SPRINZ), Auckland University of Technology, Auckland 1010, New Zealand

**Keywords:** synthesising video images, pose estimation, machine learning, biomechanics, 3D motion data

## Abstract

To increase the utility of legacy, gold-standard, three-dimensional (3D) motion capture datasets for computer vision-based machine learning applications, this study proposed and validated a method to synthesise two-dimensional (2D) video image frames from historic 3D motion data. We applied the video-based human pose estimation model OpenPose to real (in situ) and synthesised 2D videos and compared anatomical landmark keypoint outputs, with trivial observed differences (2.11–3.49 mm). We further demonstrated the utility of the method in a downstream machine learning use-case in which we trained and then tested the validity of an artificial neural network (ANN) to estimate ground reaction forces (GRFs) using synthesised and real 2D videos. Training an ANN to estimate GRFs using eight OpenPose keypoints derived from synthesised 2D videos resulted in accurate waveform GRF estimations (r > 0.9; nRMSE < 14%). When compared with using the smaller number of real videos only, accuracy was improved by adding the synthetic views and enlarging the dataset. The results highlight the utility of the developed approach to enlarge small 2D video datasets, or to create 2D video images to accompany 3D motion capture datasets to make them accessible for machine learning applications.

## 1. Introduction

Since the emergence of biomechanics as a modern scientific discipline, high-quality, multi-scale analysis of human motion has been restricted to laboratory environments. For the sports biomechanist, gold-standard precision and accuracy comes from laboratory-grade three-dimensional (3D) data. This data is typically generated by a retro-reflective motion capture system (multiple near-infrared cameras that optically detect spherical markers affixed to an athlete’s body) to record 3D kinematics, predominantly in conjunction with ground-embedded force plates to directly measure ground reaction forces (GRFs) and calculate joint kinetics [1]. In contrast to clinical biomechanists, who in addition to motion capture and force plate data also collect two-dimensional (2D) video data for clinical review, sports biomechanists have not traditionally prioritised concurrent collection of 2D video. This is explained by various factors, not the least the competing demands—particularly during the early evolution of these technologies—of recording high-speed 2D video (with high shutter and light requirements), while simultaneously conducting 3D motion capture (which has historically demanded low lux environments). Along with historical hardware constraints surrounding multi-camera synchronisation, as well as data transmission and storage capacity, the costs associated with 2D video collection generally eclipsed its utility.

As a product of this history, sports biomechanists are awash with legacy datasets comprising 3D motion and force plate data, with limited accompanying 2D video. These datasets are either large banks of commonly performed movements, such as running [2,3] or sidestepping/cutting manoeuvres [4,5], or smaller, targeted collections on specific movements or populations, such as curve sprinting [6], long jumping [7], cricket bowling [8,9], field hockey flicking [10] and rugby scrummaging [11]. Beyond being a quirk of history, the absence of 2D video from 3D datasets has been brought into sharp relief due to heightened interest in the application of computer vision-based machine learning techniques to sports biomechanics; techniques that often depend critically on the availability of large volumes of 2D video image frames. This historic absence—and accompanying contemporary need—motivated the novel solution proposed in this paper.

While laboratory-grade 3D data offers gold-standard accuracy and reliability for technique analysis [12,13], laboratory environments do not easily allow for the assessment of other contributing factors to athletic performance (e.g., game-based fatigue, attentiveness, opponents, noise and tactics). Additionally, the requirement to contact a ground-embedded force plate [6,7,10], while affixed with 50–100 markers [6,7,9,10] is likely to impact an athlete’s behaviour and performance during lab-based assessments [14]. The trade-off between laboratory-grade accuracy and field-based ecological validity has accelerated research to shift applied sports biomechanics data collection practices from the lab to the field [15].

To facilitate this lab-to-field shift, wearable sensors and video-based motion analysis are increasingly popular tools, given their relative ease-of-use, low implementation costs [16] and suitability for competitive environments. Being non-optical, body-attached wearable sensors do not suffer from occlusion, but they have the disadvantages in that they can physically obstruct movement [17,18] and that they present safety risks, especially in impact sports [19]. Wearable sensors also report varying data accuracy and reliability profiles depending on the environment and use-case [20,21,22]. Two-dimensional video-based analysis presents an attractive alternative, as it overcomes the physicality of wearable sensors and permits data collection at a distance to both athletes and field of play. Consumer-grade video capture tools are increasingly prevalent in both competition and training environments [18], with adjustable sampling rate high-resolution cameras now available in most vision-enabled smartphones and tablets. A downside of ubiquitous access to video recording is a lack of standardisation in camera recording angle, either as a function of operator location (requiring panning) or camera position restrictions (imposed by sites or sports governing bodies) [23]. This limits the utility of collected video. A further, more systemic downside of video-based monitoring and analysis is that it expedites and normalises surveillance [24]; specifically, athlete surveillance, often in the absence of necessary conversations regarding the purposes of collection and associated athlete protections and rights [25]. In removing the encumbrances of body-attached wearable sensors or the protocols of a laboratory setting, it is imperative not to lose essential processes around voluntary, informed consent and other legal and ethical commitments that those environments necessitate.

Computer vision-based machine learning tools, such as human pose estimation models that are used to auto-identify body locations (keypoints), increasingly offer labour-saving opportunities for sports biomechanists who wish to undertake on-field and in-competition video analysis. Pose estimation models were originally trained using large image databases with manually-annotated keypoint information (digitised by non-biomechanists) to identify body locations in 2D images [26]. These models have been used with a reasonable level of accuracy for 2D on-field analysis (differences of 10–30 mm when referenced to the 3D location of the same keypoint) [27]. Based on this information, joint angles can be calculated, noting dependency on the quality of the original input data and the camera recording angle with respect to the plane of motion being analysed (i.e., camera view should be orthogonal for 2D planar analysis) [28].

A particular challenge for field-based assessments when compared to laboratory assessments, is the impracticality of instrumenting the player-surface interaction for direct recording of GRFs, either through ground-embedded force plates in playing surfaces or shoe-mounted sensors. This has led researchers to develop alternative approaches to estimate GRFs, such as applying analytical models [29,30], or machine learning models trained using different data types as inputs, including motion trajectories [5,14,31], joint angles [14,32] or inertial sensor data [31,33,34]. Very recently, early efforts to estimate 3D GRFs using a particular kind of deep machine learning model, an artificial neural network (ANN), using 2D pose estimation keypoints have been published [35]. While promising, this approach relies on a large dataset of high-quality, concurrently collected and synchronised 3D force and 2D video data. If sports biomechanists are to further leverage the potential of computer vision-based machine learning techniques beyond the otherwise limiting factors of dealing with small, highly-specific populations and actions—as are endemic in elite and high-performance sport—this is likely to depend on extensive libraries of 2D video data, well beyond those that presently exist.

The aim of this paper is to present a novel method and validate a workflow to synthesise 2D video from de-identified 3D motion data using an animated 3D human body shape, or hull. The utility of this method is tested and demonstrated by using a synthesised video dataset to estimate GRFs. Finally, the paper highlights technical, as well as broader societal considerations, in repurposing 3D data to synthesise and enlarge 2D video datasets.

## 2. General Design

**Figure 1:**
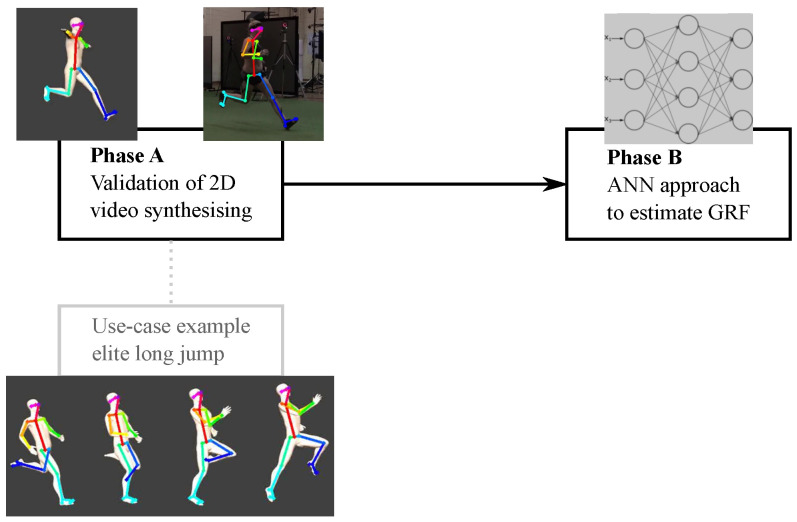
Design overview of the study.

This study was undertaken in two phases (Figure 1). In Phase A, we developed and validated a workflow for synthesising 2D video image frames from 3D motion data trajectories of sidestepping manoeuvres. We further outlined a use-case example for synthesising 2D video from 3D data trajectories for a more complex, sport-specific task of elite long jumping. In Phase B, to test the efficacy of the synthesised video approach, we trained an ANN to estimate GRFs from synthesised videos. To validate the output, we tested the ANN trained on synthesised videos using: (1) synthesised videos and (2) recorded (real) videos as inputs. ANN-derived GRF estimates from both video conditions were compared against ground-truth force plate recorded GRF data.

## 3. Phase A Methods and Results

### 3.1. Development and Validation of a Novel 2D Video Synthesis Method

#### 3.1.1. Dataset

Unplanned 45° sidestepping trials, collected from three professional and semi-professional female Australian Rules Football Players (171 cm, 57 kg; 169 cm, 58 kg; 165 cm, 65 kg), were used to validate the method of synthesising videos (selected 2D video camera views) from 3D motion capture data. Real recorded 2D video data and 3D motion capture data were collected in The University of Western Australia’s (UWA) Biomechanics laboratory and the study approved by the University’s Human Ethics Committee (RA/4/1/2593). All participants provided written consent for data collection, analysis and publication.

**Figure 2:**
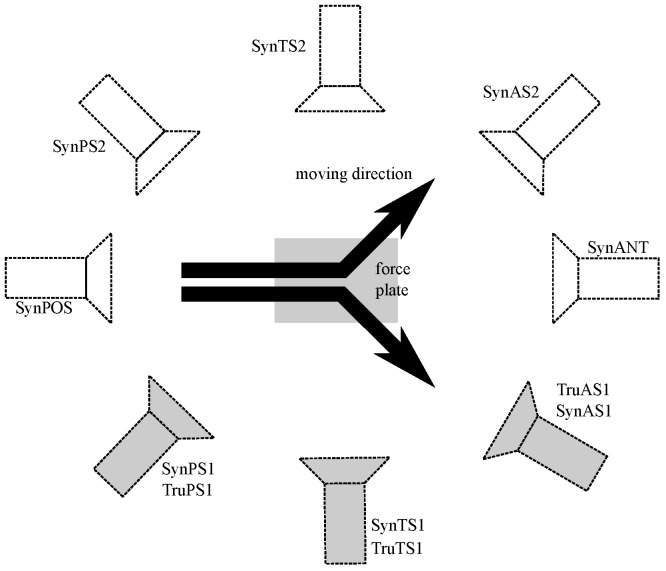
Set-up of the 2D video cameras during motion capture (grey), and eight additional synthesised camera views (white). During data collection, three in situ real cameras were positioned on the right side of the force platform in posterior-sagittal (TruPS1), true-sagittal (TruTS1), anterior-sagittal (TruAS1) positions with respect to the force plate and movement direction of the participant. The locations of the three in situ camera positions were also synthesised (SynPS1, SynTS1, SynAS1), along with an additional five synthesised camera views: posterior-sagittal (SynPS2), true-sagittal (SynTS2) and anterior-sagittal (SynAS2) on the left side of the force plate, with one positioned directly anterior (SynANT) and one posterior (SynPOS) to the force plate and the participant direction of travel. i.e. real cameras on the right side were synthesised on both the right and the left sides of the force plate. The 23 × 3D Vicon system cameras are not displayed.

Three-dimensional motion trajectory data were collected using a 23-camera VICON System (VICON, T40, Oxford, UK, 200 Hz) with GRF data recorded using a 1200 × 1200 mm force plate (Advanced Mechanical Technology Inc., Watertown, MA, USA, 2000 Hz). Each participant was affixed with 43 retro-reflective markers in accordance with the UWA lower body and trunk marker-set [36,37]. As the data used in the present study was collected as part of a larger project to validate lower limb computer vision machine learning models, the 3D dataset comprised only trunk and lower limb markers but no upper arm, forearm or hand markers. Participants approached the force plate at a speed of 4.5–5.5 m s^−1^ determined by timing gates (SmartSpeed Pro, Fusion Sports, QLD, Australia) positioned 3 m and 0.5 m in front of the force plate in the approach runway. The desired sidestepping direction (left, right) or a dummy straight run trial (used to prevent the participant anticipating sidestepping direction), was indicated by an arrow displayed on a projector screen, which was triggered as the participant crossed the timing gates 0.5 m immediately prior to force plate contact. In every captured 3D motion capture trial, three 2D video cameras (Sony HDR-CX700, 25 Hz, 1920 × 1080 px) recorded footage from three locations (Figure 2), sagittal to the plane of movement; slightly posterior to true sagittal camera location (TruPS1), true sagittal location (TruTS1), slightly anterior to true sagittal location (TruAS1). The TruPS1 and TruAS1 cameras were panned approximately 30°, such that the central location of the camera field of view was the force plate, where the sidestepping manoeuvre was initiated. All three 2D camera fields of view were positioned to align with the volume directly above the force plate, where the participant stance phase for the sidestepping or straight run occurs.

#### 3.1.2. Synthesising 2D Videos

**Figure 3:**
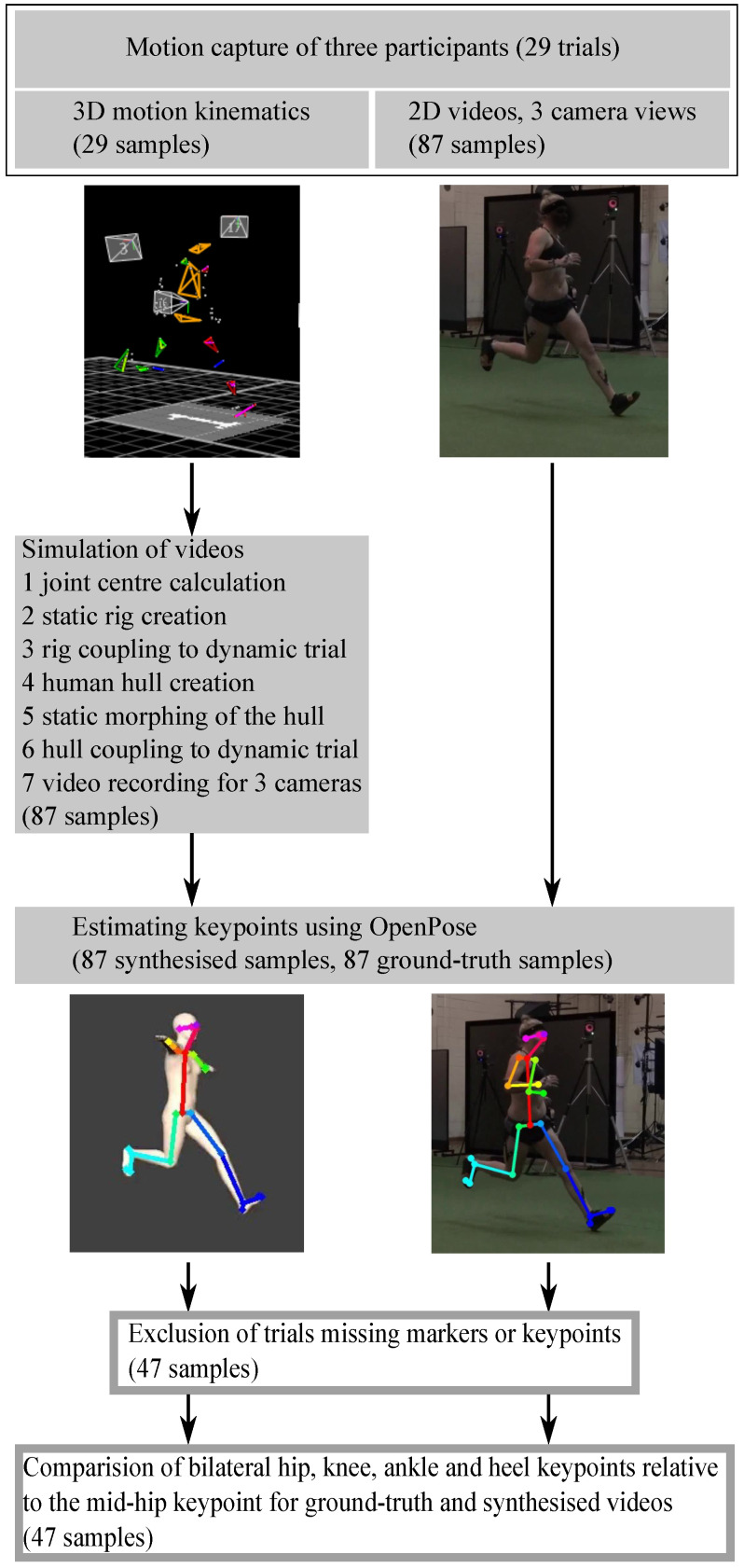
Design overview of the synthesised 2D video method validation study; 3D motion trajectory data from three participants was used for validation. Synthesised 2D videos showing errors identified by visual inspection, such as body segments following unachievable movement trajectories, or those missing pose model estimated keypoints were excluded.

A seven-step Python (Python Software Foundation, v.3.9) framework was developed to automatically synthesise 2D video images from 3D motion trajectory data (Figure 3).

**Figure 4:**
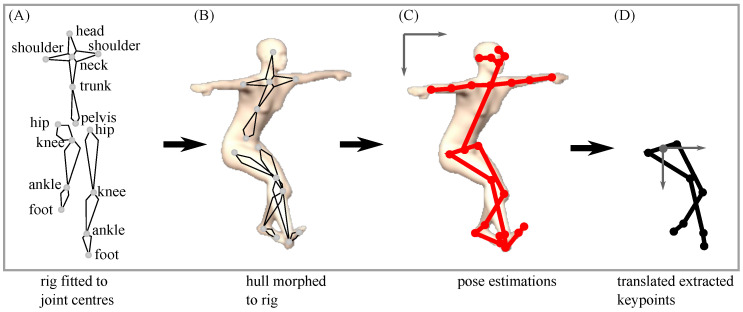
(**A**) A 3D animation rig was fitted to body segment and joint centres calculated from the participant-specific 3D motion capture static trial data. (**B**) A female 3D hull was morphed to the rig, and (**C**) 25 anatomical/segment 2D OpenPose keypoints were estimated. The arms of the 3D hull remained in a fixed neutral T-pose posture as the fitted rig did not contain arms due to the absence of arm 3D markers. (**D**) Eight lower limb keypoints identified by OpenPose, which returned a high detection rate, were extracted and translated to a moving pelvis coordinate system.

(1) Using the affixed 3D retro-reflective markers, segment and joint centres (or proxy representations) for the head, neck, shoulder (glenohumeral), trunk, pelvis and bilateral hip, knee, ankle and foot, were determined using custom UWA biomechanical models [36,37]. The head centre was defined as the midpoint of the four head markers, the neck as the midpoint of the C7 and clavicle markers, the shoulder joint centre from three markers positioned about the shoulder as per the intersection method of Campbell et al. [38], the upper body as the midpoint of T10 and sternum marker, the pelvis centre as the midpoint of the four pelvis markers. The hip joint centre was defined according to Harrington et al. [39], the knee joint centre as the midpoint of the medial and lateral epicondyle markers, the ankle joint centre as the midpoint of the medial and lateral malleoli and the foot as the midpoint of the markers at the first and fifth metatarsal heads. Upper limb segment (upper arm, forearm, hand) and joint centres (elbow, wrist) were not estimated due to the absence of 3D markers for this purpose.

(2) A 3D animation rig was fitted to the segment and joint centres in a static 3D motion capture trial using the animation software Blender (v.2.79, Blender Foundation, Amsterdam, the Netherlands) which has a Python API that enables automatic rig creation. In animation software, *rigging* refers to the process of creating the bone structure of a 3D model. This bone structure is necessary to manipulate the 3D model as if it were a puppet. Individual bone lengths were determined using segment and joint centre locations.

(3) The rig was coupled with the dynamic (moving) 3D motion trials using built-in constraints. These constraints define the movement of individual segments that build the rig. Blender’s custom *damped track* constraints account for small differences in the distance between markers that define a bone such that segment lengths remain constant. *Copy location* constraints serve to couple bone pairs (e.g., thigh and shank to build the knee joint) (Figure 4A).

(4) A female 3D human hull (body shape) was created in MakeHuman (MakeHuman Community, v.1.2.0) with the following dimensions: height 159 cm, chest circumference 82.5 cm, waist circumference 71.2 cm and hip circumference 94.2 cm.

(5) The 3D hull was imported into Blender software using Python. For accuracy, the pose of the 3D hull and the pose of the participant as recorded in the 3D static motion capture trial were required to be generally aligned (with respect to a common global coordinate system, inclusive of a standard distance between feet). Alignment was achieved via iterating translation and rotation of the static motion capture data until a suitable fit was achieved with the 3D hull, as determined by visual inspection. As the 3D motion capture data collected in this study was part of a larger project, the static 3D standing calibration trials were not initially captured with the intention of fitting a 3D human hull to the data, making the alignment step necessary, as the 3D hull itself could not be manipulated without first fitting a rig.

(6) After fitting the 3D motion capture static trial to the 3D hull, the resulting rigged hull was coupled to all dynamic sidestepping trials (Figure 4B).

(7) The resulting 3D animation of the sidestepping trial fitted with a 3D female human hull, facilitates a near infinite number of 2D camera views able to be recorded from within the software. To validate the synthesised video workflow, three synthesised video views (SynPS1, SynTS1, SynAS1) were created, which corresponded with the real Sony video camera locations (TruPS1, TruTS1, TruAS1) used during live data capture (Figure 2). A total of 47 synthesised 2D videos were exported from the 3D hull sidestepping trial views at a resolution of 960 × 540 px and a frame rate of 100 Hz.

#### 3.1.3. 2D Pose Estimation Using OpenPose

OpenPose [40], a real-time human pose detection library, was used to estimate 25 keypoints (nose, neck, mid-hip, bilateral eyes, ears, shoulders, elbows, wrists, hips, knees, ankles, heels, big toes and small toes) in the 47 synthesised video view images and the 47 real 2D video images (Figure 4C). As the initial 3D sidestepping trials (and subsequently the synthesised 2D videos) did not include upper limb marker trajectories, and given previous research shows that lower limb marker trajectories alone are sufficient to estimate ground reaction force [14], only bilateral heel (calcaneus), ankle, knee and hip keypoint outputs from the synthesised versus real videos were compared. The toe (metatarsal) keypoints returned a low detection rate in both the real and synthesised videos and were subsequently excluded from analysis. To standardise the keypoint outputs to aid comparison, all synthesised and real 2D key point output locations in each video were translated and reported relative to the moving coordinate system of the pelvis (Figure 4D).

#### 3.1.4. Data Processing

The eight OpenPose keypoints were filtered using a moving average filter with a window size of five frames. To account for differences in video frame rate and resolution between the real 2D video (25 Hz, 1920 × 1080 px) and synthesised 2D videos (100 Hz, 960 × 540 px), all stance phases (6–10 frames in real videos, 24–40 frames in synthesised videos) were time-normalised to 100 frames and the keypoint values estimated in the real videos were divided by two to account for resolution differences. A final sanity check was performed by visually inspecting the 2D synthesised videos. The final dataset for the Phase A synthesised 2D video validation study contained eight OpenPose keypoint outputs for each of the 47 real videos and its 47 matched equivalent synthesised 2D videos (Figure 3). To compare differences in keypoint output location between the real versus synthesised 2D videos, the Euclidean distance between matched keypoints in all matched video frames was determined.

#### 3.1.5. Results

**Figure 5:**
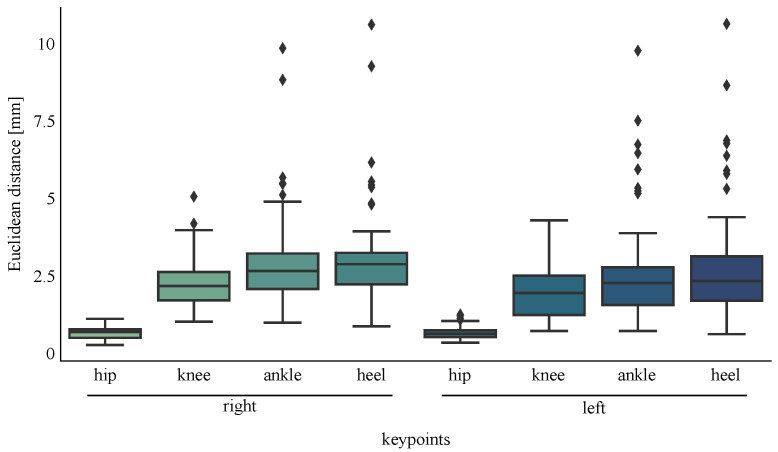
Euclidean distance distribution between the keypoints estimated in three synthesised versus real videos (95% confidence interval) and three real camera locations: right side anterior-sagittal, true-sagittal and posterior-sagittal to the participant’s forward direction plane of motion (TruPS1, TruTS1, TruAS1, SynPS1, SynTS1, SynAS1).

The Euclidean distance (ED) between the eight selected keypoints estimated from the real and synthesised videos is shown in Figure 5. The smallest ED (<0.8 mm) between the same keypoint locations in real versus synthesised videos was found for the left and right hip keypoints (mean distance right hip 0.74 mm, left hip 0.76 mm). Interestingly, although the ED between the remaining keypoints remained small (<4 mm), ED progressively increased as the keypoint location moved distally away from the pelvis (right knee 2.49 mm, left knee 2.11 mm, right ankle 3.34 mm, left ankle 3.01 mm, heel right 3.49 mm, left 3.21 mm).

### 3.2. Elite Long Jump Data Applied Use-Case

The use of 3D motion capture data to generate 2D synthesised videos opens a range of novel opportunities to re-use sparse de-identified 3D motion capture data. A working example of a small complex dataset that could benefit from such an approach is that of elite long jumpers collected at the German Sport University Cologne. The study was approved by the University’s Ethics Committee (approval number: 040/2016) and all participants provided informed written consent. The dataset contained eleven long jump trials recorded from five athletes (three trials of athlete 1, one trial of athlete 2, two trials of athlete 3, four trials of athlete 4 and one trial of athlete 5) [7].

Anthropometric data from each athlete (height, length, width and/or circumference of feet, shanks, thighs, torso, arms, neck and head) were measured prior to 3D motion capture and used to create personalised human 3D hulls in MakeHuman. The 3D motion capture trajectory data (VICON, Oxford, UK, 20 cameras (MX40), 250 Hz) contained the final three steps of the run-up (approach), the take-off and the initial swing of the flight phase. Once again, the rigged 3D human hulls can be captured from any camera view from within the 3D software, and 2D pose estimation models were then used to obtain keypoint locations. As no real 2D video data were available, no additional validation of the synthesised videos was possible. However, this use-case example shows that the proposed synthesised video view pipeline is transferable to more complex sport-specific movements, facilitating a near infinite number of synthesised 2D camera views from the original 3D marker trajectory data.

**Figure 6:**
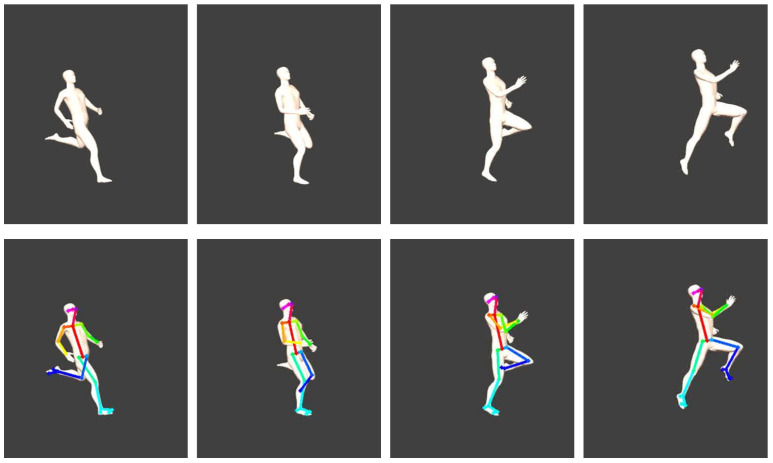
Single frames of one synthesised video of a long jump trial (**top**) with estimated OpenPose outputs from the synthesised video views (**bottom**).

Figure 6 displays 2D pose estimation keypoints identified in the synthesised videos. The motion of the long jumper was successfully animated and 2D keypoint detection using OpenPose was feasible using the synthesised 2D camera views recorded from within the software.

## 4. Phase B Methods and Results

### 4.1. Phase B: Training and Validating an ANN for Ground Reaction Force Estimation Using Synthesised Video Images

#### 4.1.1. Dataset

**Figure 7:**
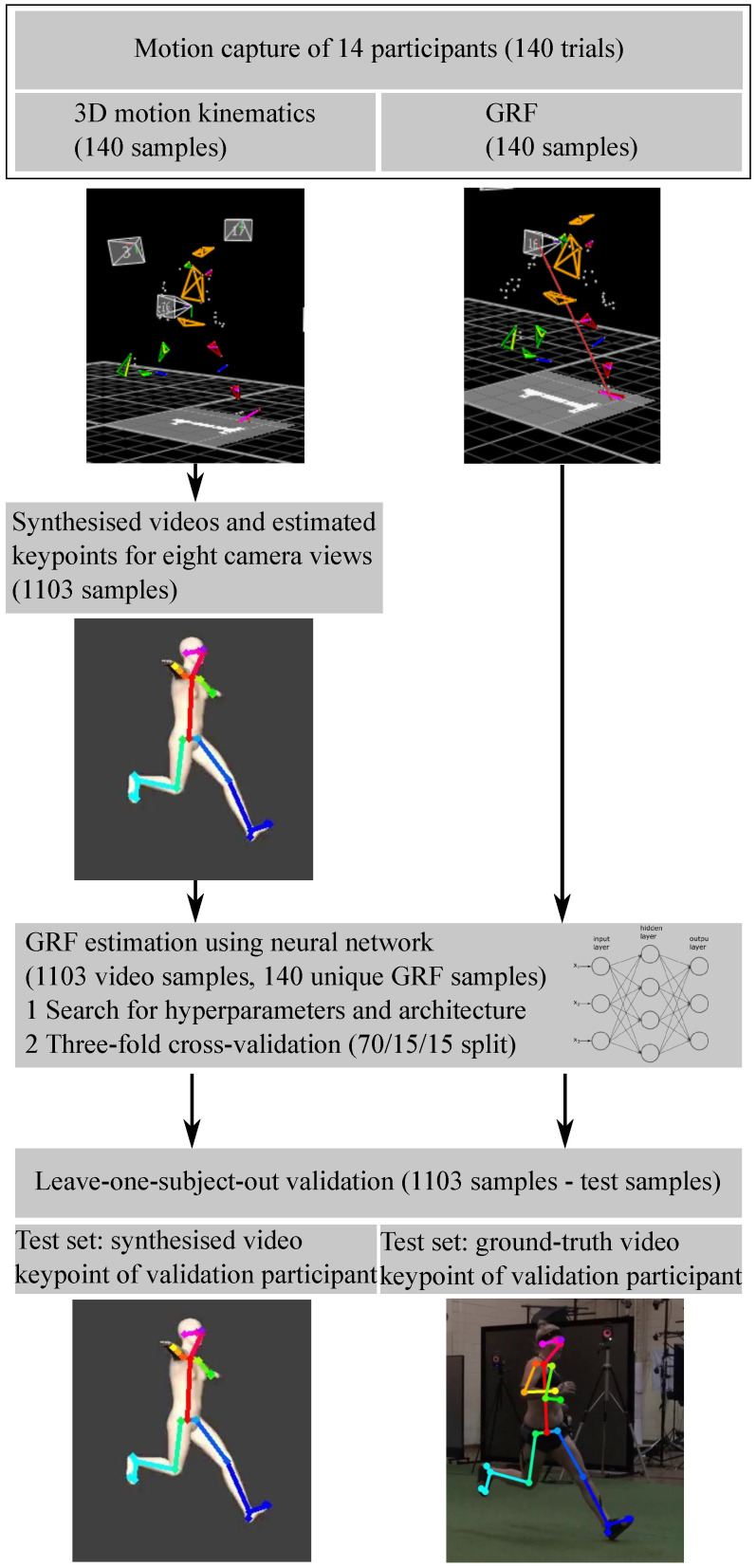
Design overview of the machine learning application to estimate ground reaction forces using synthesised 2D video data.

The full Phase B dataset comprised unplanned 45° sidestepping trials, collected from 14 professional and semi-professional female Australian Rules Football Players (23 ± 3.74 years, 62.77 ± 5.41 kg, 168 ± 4 cm). Data were collected in The University of Western Australia’s Biomechanics laboratory and the study approved by the University’s Human Ethics Committee (approval number: RA/4/1/2593). All participants provided written consent for data collection, analysis and publication. Three-dimensional motion trajectory, GRF and 2D video data were collected as described in Phase A Section 3.1.1. Using the approach outlined in Phase A Section 3.1.2 synthetic 2D videos generated from the fields of view of eight virtual cameras were also recorded (inside the software). Three of the eight synthesised views (SynPS1, SynTS1, SynAS1) corresponded with the real in situ Sony video camera locations (TruPS1, TruTS1, TruAS1), with an additional five synthesised 2D views surrounding the volume (SynPOS, SynPS2, SynTS2, SynAS2, SynANT) (Figure 2). A total of 1103 synthesised 2D videos from eight virtual camera views were exported from the software at a resolution of 960 × 540 px and a frame rate of 100 Hz. Similar to Phase A Section 3.1.3, OpenPose was used to estimate 25 keypoints in all synthetic videos with the eight previously validated keypoints used for further analysis; bilateral heel (calcaneus), ankle, knee and hip locations. All keypoints were translated and referenced to the pelvis to enable between video condition comparisons (Figure 7).

#### 4.1.2. 2D Video Data and 3D GRF Data Processing

GRF data were filtered using a moving average filter with a window size of ten frames and normalised to each participant’s body weight. The OpenPose keypoints were filtered using a moving average filter with a window size of five frames. GRF and keypoint time-series data across the stance phase were time-normalised to 100 frames. The dataset used for ANN training contained 1103 synthesised videos from eight camera views (SynPS1, SynTS1, SynAS1, SynPOS, SynPS2, SynTS2, SynAS2, SynANT) of 14 participants and 140 unique GRF samples (Figure 7).

#### 4.1.3. ANN Model Training

A multi-layer perceptron neural network, as the classical and simplest class of ANN [41], was trained to estimate the GRF based on the keypoint time-series estimated in the 1103 synthesised videos. Since multi-layer perceptron neural networks cannot model time-dependencies, the input and target data were required to be flattened, which resulted in an input matrix of size [n samples × (8 keypoints × 2 dimensions (u and v) × 100 frames)] = [n samples × 1600 features] and an output matrix of size [n samples × (GRF × 3 dimensions (medio-lateral, anterior-posterior, vertical) × 100 frames)] = [n samples × 300 features].

#### 4.1.4. ANN Validation

Two validation sub-studies were performed using real and synthetic 2D videos as input data (Figure 7).

First, the ANN performance for estimating GRFs using 2D OpenPose keypoints was investigated. For this purpose, an automated hyperband search [42] was performed to find the architecture (number of layers, number of neurons per layer) and hyperparameters (learning rate, dropout, activation function) resulting in the smallest loss. It was ensured that none of the identified parameters equalled the upper or lower boundaries of the search. The dataset was split into a 70% training, 15% validation and 15% test set with no participant being part of more than one dataset (subject-wise split) [43]. A three-fold cross-validation was performed to ensure that the architecture and hyperparameters found resulted in good overall results with no overfitting.

Second, the accuracy of the GRF estimation when using the 2D keypoints from synthesised videos versus 2D keypoints from real videos were compared using Phase A participant data as the test data (Figure 7). For this purpose, a leave-one-subject-out (LOSO) validation was undertaken for the three participants’ data from Phase A. The ANN model was trained on all synthesised data besides the left out participant. The resulting three trained models were then tested using (1) the synthesised video and (2) real video, keypoints of the left out participant.

In both sub-studies, GRF estimation accuracy was evaluated using the root-mean-square error normalised to the range of the data (nRMSE) and the correlation coefficient for a 95% confidence interval. We considered our developed approach of enlarging existing small video datasets using synthesised videos to estimate GRFs valid if the synthesised and real OpenPose keypoints nRMSE < 2% and the correlation coefficient > 0.9 [44].

#### 4.1.5. Results

**Figure 8:**
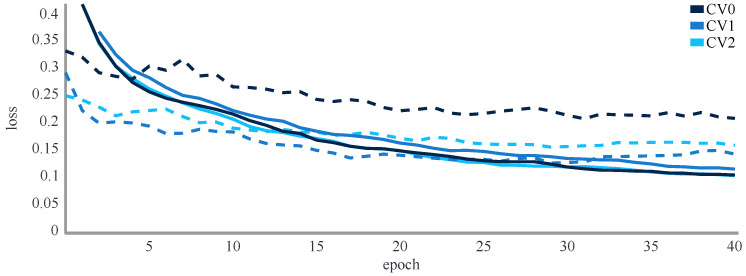
Training loss (solid line) and validation loss (dashed line) of the loss during the 3-fold cross validation to find the optimum hyperparameters. No overfitting observed.

In the first step, the hyperband search resulted in a neural network architecture with two hidden layers comprising 3000 and 2000 neurons. The optimal initial learning rate was 0.0003, dropout rate 0.4 and the best activation function was a rectified linear unit. The loss curves of the three validation runs (Figure 8) showed no overfitting during training for 40 epochs. The training loss, as a measure of how well a connection between inputs and outputs can be established, continuously decreased, while the validation loss clearly decreased during the first training epochs and only minimally decreased as the run time extended. Implementing early-stopping and thereby training for fewer epochs resulted in reduced accuracy for the test set. Subsequently, all networks were trained for 40 epochs.

The cross-validation resulted in good accuracy and low variation between the three validation runs. The mean correlation coefficient between ground-truth recorded and estimated 3D GRF was 0.954 ± 0.005 (medio-lateral GRF 0.944 ± 0.005, anterior-posterior GRF 0.963 ± 0.005, vertical GRF 0.955 ± 0.004) and the nRMSE between ground-truth and estimated 3D GRF 13.30 ± 0.46% (medio-lateral GRF 17.09 ± 0.910%, anterior-posterior GRF 11.96 ± 0.53%, vertical GRF 10.85 ± 0.28%).

**Figure 9:**
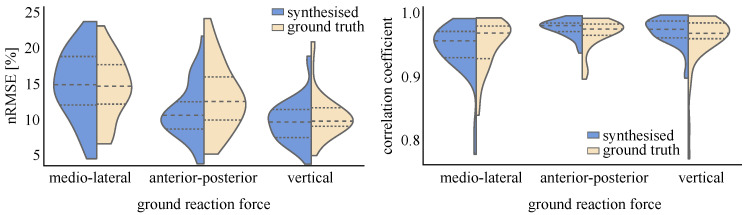
nRMSE (**left**) and correlation coefficient (**right**) distributions for estimated 3D ground reaction forces (95% confidence interval). The dashed horizontal lines present the median value and the 25th and 75th percentiles.

The results of the second sub-study, the LOSO-validation, are displayed in Figure 9. Testing the ANN using synthesised 2D videos resulted in a mean correlation coefficient between ground-truth and estimated 3D GRF of 0.949 (medio-lateral GRF 0.931, anterior-posterior GRF 0.963, vertical GRF 0.954) and mean nRMSE between ground-truth and estimated 3D GRF of 12.82% (medio-lateral GRF 16.22%, anterior-posterior GRF 10.79%, vertical GRF 11.48%). Using real videos as input resulted in a mean correlation between ground-truth and estimated 3D GRF of 0.943 (medio-lateral GRF 0.926, anterior-posterior GRF 0.957, vertical GRF 0.948) and mean nRMSE between ground-truth and estimated 3D GRF of 13.55% (medio-lateral GRF 17.61%, anterior-posterior GRF 11.11%, vertical GRF 11.94%). An overall decrease in accuracy (0.73%) was observed when using the keypoints estimated from real videos. The medio-lateral GRF component was the most greatly affected with an observed nRMSE increase of 1.40%, with the anterior-posterior and vertical GRF estimations reporting only small differences, 0.32% and 0.46%, respectively.

**Figure 10:**
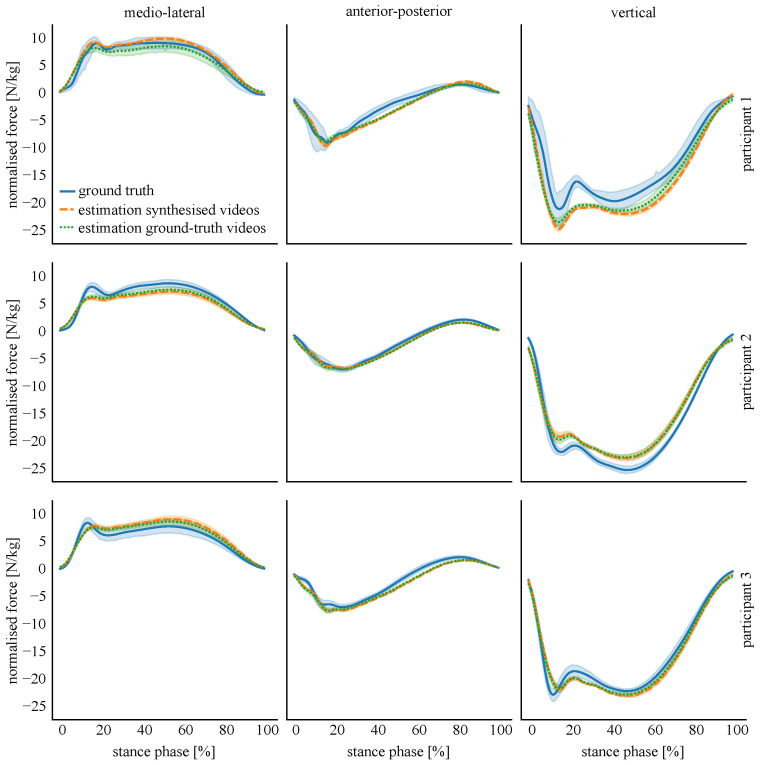
Estimation of the single GRF components for the three validation participants of Phase A. The solid lines represent the mean value and the shaded area the standard deviation.

Figure 10 shows the estimated GRF for the three validation participants of Phase A using keypoints estimated in the real and synthesised videos. Irrespective of video input condition (real or synthesised), the pose estimation keypoints identified in each performed equally well across participants.

## 5. Discussion

The developed method enables the synthesis of 2D video data from arbitrary camera views using 3D optical motion capture data as input. Thereby, videos of any sport-specific movement that has been performed in a laboratory setup previously can be created. This allows us to create fields of view similar to those that can be collected in a training or competition environment or even by broadcast, and a more detailed motion analysis can be performed leveraging information from laboratory testing (as detailed in Section 4). The first aim of this study was to develop a method to synthesise 2D video image frames from historic 3D data; specifically, video views of sidestepping manoeuvres using historical motion capture trajectory data and an animated 3D human hull. The workflow to synthesise the 2D video consisted of seven steps. Only two steps could not be automated—the creation of the MakeHuman 3D model and the coupling of the 3D human hull and rig. Since a non-personalised 3D hull was used, this task was only required to be undertaken once in the current pipeline. The misalignment of the 3D static motion capture trial and 3D hull required manual adjustment of the motion capture data and, consequently, time consuming visual inspection for every participant. In the present dataset, the static posture adopted by participants was not standardised, e.g., some participants stood in a neutral A-pose and others in a T-pose, feet were placed varying widths apart, and the 3D data were captured inconsistently aligned with the laboratory’s global coordinate system. Future research should investigate automatic approaches to addressing this problem as it is likely other historical datasets will suffer the same alignment hurdles. Standardising future participant 3D data collection procedures in order to mitigate or minimise the influence of this issue is strongly recommended.

The study provides a working example of the utility of the synthesised video workflow to a small, complex, use-case of historical 3D long jump data of national level European athletes. The use of personalised 3D human hulls simplified the coupling but added an additional manual step in which each participant’s 3D model anthropometry was adjusted in MakeHuman. A workflow to automate this step should also be developed in order to efficiently enlarge 2D video databases using legacy 3D motion capture data.

The developed workflow resulted in pose-estimated keypoint location average differences between 2D synthesised and real videos of less than 4 mm. Interestingly, a trend in the keypoint location differences showed a small but progressively increasing difference as the keypoint locations moved distal to the pelvis (root segment) and closer to the ground. This may be attributed to error propagation given the origin of all keypoints’ reference coordinate system is located in the pelvis, such that the ankle and heel keypoints return the largest distance to their origin. Another likely contributing factor is the low contrast between socks/shoes and floor in the real videos, resulting in a lower accuracy in the estimated keypoint locations compared to those estimated from synthesised videos containing optimum contrast. It is also worth observing that the real (TruPS1, TruTS1, TruAs1) and synthesised camera views (SynPS1, SynTS1, SynAS1) recorded motion only from the right side of the participant yet no differences were observed between left or right limb keypoint outputs. This finding suggests that synthesised camera views were unaffected by position location around the participant and suggests that multiple synthesised camera views that fully encompass the measurement volume are not required.

The second aim of the study was to train an ANN to estimate GRFs during sidestepping tasks using eight synthesised 2D camera view recordings, comparing estimation accuracy differences of the trained ANN model outputs to both synthesised and real 2D videos. The GRF could be estimated with high accuracy (correlation > 0.9, nRMSE < 14%) based on both synthesised and real videos. Overall, the accuracy was slightly lower when GRF was estimated from real video than when estimated from synthesised video. The medio-lateral GRF component was the most greatly affected but with an increase of less than 2% this effect is not considered meaningful in practice. The results highlight the potential use of synthesised videos to bridge the gap to on-field motion analysis. The simple ANN structure chosen in this example does not allow for real-time estimation of GRF. Future work should investigate the use of recurrent neural networks to enable real-time assessment of movement.

In a previous study, Morris et al. [35] used real 2D video data to estimate GRFs using 25 keypoints. Our results showed an improved estimation accuracy, especially for the medio-lateral GRF component using only eight keypoints translated to a moving reference frame originating in the pelvis. There may be an opportunity for further improvement when additional synthesised camera views are available. Further research should also consider undertaking a sensitivity analysis to determine the optimal number of keypoints and camera views. Importantly, despite the novel method employed, the GRF estimation accuracy of this study is similar to research that has trained GRF machine learning models using wearable sensor inputs [31] or joint angle inputs [14]. However, using 2D videos to estimate GRFs is advantageous for a variety of reasons; principal among them, that it reduces the physical encumbrance to athletes by removing affixed markers or sensors. The ability to synthesise 2D video views from historical 3D motion capture datasets is also an extremely valuable approach in not only increasing small 2D video datasets but also when considering the opportunities for reanimating former elite athlete datasets in a manner that facilitates opportunity for additional insights into unique actions and techniques.

### Broader Context

The development of the method and workflow presented in this paper, and the tool that it ultimately gives rise to, occurs at a time when there is significantly increased interest in the use of video-based analytics and machine learning techniques for sporting applications. It also occurs against a backdrop rarely connected to sporting applications—namely, increased regulation of biometric monitoring technologies, such as facial, gait and voice recognition technologies, given the acute risks to privacy and autonomy that these technologies present. Given this broader context, it is incumbent on researchers developing and applying novel techniques in the field of biometric monitoring and analysis—such as those advanced in this paper—to consider the societal, legal and ethical implications of their research and how their methods and publications may be utilised within and outside the discrete context in which they are published.

Some of the advantages of the presented method are that it facilitates the re-use of historic de-identified 3D data, instead of requiring the generation of new identifiable 2D video data. Though video can be blurred, it often remains identifiable, revealing personal and sensitive information. By contrast, 3D motion capture and force data is de-identified by nature. A further advantage of the technique is that, even when non-textured, 3D hulls are personalised to an individuals’ anthropometry, they are less likely to involve personal and sensitive information that give rise to concerns about privacy and autonomy. In practical applications, these advantages may be diminished if synthesised 2D video is not used to replace real 2D video, but only to augment it. Here, it is worth noting that the facility of collecting 2D video at a distance from individuals should not diminish the importance of securing voluntary and informed consent and having regard to other legal and ethical obligations around personal and sensitive information. While video may be non-obstructive, it remains potentially intrusive and invasive to individuals and communities.

While the present study involved research protocols that secured consent to the future use of collected information in machine learning applications, this is unlikely for other historic 3D datasets, requiring practitioners to ensure that they do not enable the use of information in ways that would not have been reasonably expected by the original research participants [45]. In particular, they should ensure that the information could not be used in a way that generates commercial gain for third-parties, as this has been consistently demonstrated to disrupt individuals’ reasonable expectations as to the use of their personal information. A specific development that increases the importance of such considerations is the emergence and rapid expansion of commercial entities such as Meta Platforms (formerly Facebook), Amazon and Apple into the spheres of motion analysis and virtual representation of humans.

## 6. Conclusions

This paper outlines a novel method to synthesise 2D videos from 3D motion capture data and highlights the utility of the approach by training an artificial neural network to estimate ground reaction forces using synthesised 2D videos. The method has a range of potential applications. It enables the creation of 2D videos where only 3D motion capture data exists, as well as missing or alternate camera views. Importantly, it serves to increase the size of small video datasets to a level that enables the application of machine learning techniques. We also raise broader concerns that must form part of the wider consideration of researchers seeking to re-purpose historical motion capture datasets.

## Data Availability

The data presented in this study are available on request from the corresponding author. The data are not publicly available due to source data participant consent limitations.
